# Antibiotics for Prophylaxis of Infective Endocarditis in Pediatric Patients: Knowledge and Prescribing Practices Between Italian Dentists

**DOI:** 10.3390/antibiotics15050460

**Published:** 2026-05-01

**Authors:** Martina Barone, Giovanni Bruno, Christian Bacci, Michele Basilicata, Patrizio Bollero, Raffaella Docimo, Antonio Gracco, Alberto De Stefani, Filippo Cavallari

**Affiliations:** 1 Department of Neuroscience, School of Dentistry, University of Padova, 35128 Padova, Italychristian.bacci@unipd.it (C.B.);; 2 Department of Industrial Engineering, University of Roma Tor Vergata, 00133 Rome, Italy; 3 UOSD Special Care Dentistry, Department of Experimental Medicine and Surgery, University of Roma Tor Vergata, 00133 Rome, Italypatrizio.bollero@ptvonline.it (P.B.); 4 Dental Department, UniCamillus-Saint Camillus International University of Health Sciences, 00131 Rome, Italy; 5 Pediatric Dentistry, Department of Surgical Sciences, University of Rome Tor Vergata, 00133 Rome, Italy; 6 Department of Pharmacological Sciences, University of Padova, 35131 Padova, Italy

**Keywords:** antibiotics, pediatric dentistry, infective endocarditis, antibiotic prophylaxis

## Abstract

Background: In pediatric dental care, antibiotics are routinely prescribed for both therapeutic and preventive purposes. Their use is primarily indicated for the management of widespread dental or oral infections, in conjunction with appropriate clinical treatment. Additionally, antibiotics are administered to prevent infective endocarditis (IE) in patients identified as being at increased risk. The present study aimed to evaluate the knowledge and prescribing practices of Italian dentists regarding antibiotic use in pediatric patients through an anonymous online questionnaire. Methods: A specifically designed questionnaire was electronically distributed to a group of Italian dentists. The questionnaire included three sections: demographic data, general knowledge on antibiotic prescribing, and IE prophylaxis in pediatric dentistry. Data were analyzed using appropriate statistical methods. Results: The study included 242 Italian dentists. Only a limited number of statistically significant differences were observed between specialists in pediatric dentistry and general dentists or those with other specializations, as well as between practitioners who mainly treat pediatric patients and those who predominantly treat adults. Regarding antibiotic prophylaxis for IE, most respondents identified amoxicillin as the first-line antibiotic for pediatric patients without penicillin allergy, whereas nearly 30% indicated clindamycin for patients with penicillin allergy. The knowledge about the dosage of assumption of the antibiotic of choice and the timing of administration of the antibiotic prophylaxis were considered as not sufficient. Conclusions: Important gaps remain in dentists’ knowledge of current guidelines for IE prophylaxis, particularly regarding drug dosage and administration. Increased awareness of updated recommendations and potential adverse effects of alternative antibiotics, such as clindamycin, is needed.

## 1. Introduction

In pediatric dental practice, antibiotics are used for both treatment and preventive purposes. Their therapeutic use is primarily indicated for the management of widespread dental or oral infections and should be combined with the most appropriate clinical procedure for each case. Moreover, antibiotics are administered for the prevention of infective endocarditis (IE) in patients at increased risk of developing the condition and of experiencing complications related to their underlying medical status. In these cases, prophylaxis is administered prior to selected invasive dental procedures to reduce or eliminate transient bacteremia. Antimicrobial resistance, whose emergence is closely linked to the increasing use of antibiotics in both medical and dental settings, represents a major global health concern. Insufficient awareness of appropriate indications for antibiotic prescribing is a key factor contributing to their overuse and misuse [1,2].

The main objective of this cross-sectional survey was to evaluate the knowledge and prescribing behaviors of Italian dentists regarding antibiotic use in pediatric patients, both for the supportive management of dental or oral infections and for the prevention of infective endocarditis (IE). A secondary objective was to compare these aspects between pediatric dentistry specialists and general dentists or other practitioners involved in the care of pediatric patients. The present article focuses on the findings related to antibiotic prophylaxis for IE in pediatric dentistry, while the results concerning antibiotic use for the treatment of dental or oral infections have been reported in a previous publication. The questionnaire used in the present study was administered together with the one used in our previous publication on antibiotic use for dental or oral cavity infections in pediatric dentistry, from which a different subset of results was analyzed and reported separately.

## 2. Results

### 2.1. Study’s Sample Size and Description

The study population consisted of a convenience sample of 242 Italian dentists, with a response rate of 34.6%. The sample was predominantly composed of women (68%), practitioners from Northern Italy (70%), dentists with 20 years or less of professional experience (approximately 70%), general practitioners (58%), clinicians treating both adults and pediatric patients (57%), and dentists working in the private sector (77%). The main characteristics of the sample are summarized in Table 1.

### 2.2. Prophylaxis of Infective Endocarditis—Clinical Conditions

Overall knowledge regarding the use of antibiotics for the prophylaxis of infective endocarditis in specific clinical conditions is presented in Table 2 for the entire sample, while detailed subgroup analyses (based on experience, academic level, patient population, and practice setting) are provided in the Supplementary Materials (Tables S1–S4).

### 2.3. Prophylaxis of Infective Endocarditis—Dental Procedures

Overall knowledge regarding the use of antibiotics for the prophylaxis of infective endocarditis prior to specific dental procedures is presented in Table 3 for the entire sample, while subgroup analyses (based on experience, academic level, patient population, and practice setting) are reported in Tables S5–S8 in the Supplementary Materials.

### 2.4. Knowledge on the Antibiotic of First Choice for the Prophylaxis of IE in Pediatric Patients with No Allergy to Penicillins

Information on the antibiotic of first choice for the prophylaxis of infective endocarditis in patients without penicillin allergy is presented in Table 4 for the overall sample, while subgroup analyses (based on experience, academic level, patient population, and practice setting) are reported in Tables S9–S12 in the Supplementary Materials.

### 2.5. Knowledge on the Antibiotic of First Choice for the Prophylaxis of IE in Pediatric Patients with Allergy to Penicillins

Information on the antibiotic of first choice for the prophylaxis of infective endocarditis in patients with penicillin allergy is presented in Table 5 for the overall sample, while subgroup analyses (based on experience, academic level, patient population, and practice setting) are reported in Tables S13–S16 in the Supplementary Materials.

## 3. Discussions

The prudent and appropriate use of antibiotics is crucial to minimize adverse drug reactions and reduce the emergence of antimicrobial resistance. The aim of this study was to investigate, through a structured questionnaire, the knowledge and prescribing practices of Italian dentists regarding antibiotic use in pediatric patients. The findings highlight several gaps in knowledge and prescribing practices related to antibiotic prophylaxis for infective endocarditis in pediatric dentistry. Despite the availability of clear international guidelines, the variability in responses suggests incomplete adherence and a degree of uncertainty in clinical decision-making.

IE is an infection of the endocardium, the inner lining of the heart, or of the heart valves, most commonly caused by bacterial colonization and, less frequently, by other pathogens such as fungi [3]. The microorganisms most often involved include viridans group streptococci, *Staphylococcus aureus*, and enterococci. Collectively, these pathogens account for approximately 80–90% of cases, while *Staphylococcus aureus* alone is responsible for about 30% of cases in developed countries. Fungal infections are uncommon, representing roughly 1% of all cases of IE [3,4,5].

IE is a rare yet potentially fatal condition. Despite improvements in diagnostic methods, medical treatment, surgical approaches, and complication management, it continues to be associated with considerable morbidity and mortality. A multicenter study conducted between 2003 and 2010 in pediatric hospitals in the United States reported an incidence of IE-related hospitalizations ranging from 0.05 to 0.12 cases per 1000 hospitalizations [4,5,6].

The rationale for antibiotic prophylaxis in infective endocarditis is to reduce or eliminate transient bacteremia associated with invasive dental procedures [4,7,8]. Since 1955, the American Heart Association (AHA) has issued recommendations for the prevention of IE [6].

According to the most recent American Heart Association (AHA) guidelines (2021) [9], antibiotic prophylaxis is recommended for patients with valvular heart disease who present one or more of the following clinical conditions:

- Prosthetic cardiac valves, including transcatheter-implanted prostheses and homografts
- Prosthetic material used for cardiac valve repair, such as annuloplasty rings, chords or clips
- Previous IE
- Unrepaired cyanotic congenital heart defect (CHD) or repaired CHD, with residual shunts or valvular regurgitation at the site of or adjacent to the site of a prosthetic patch or prosthetic device
- Cardiac transplant recipients with valve regurgitation due to a structurally abnormal valve.

Furthermore, the AHA guidelines indicate that antibiotic prophylaxis is appropriate before dental procedures involving manipulation of gingival tissue, the periapical region of the teeth, or perforation of the oral mucosa. Beyond the conditions listed above, antibiotic prophylaxis for infective endocarditis is not recommended for other cardiac conditions or dental procedures.

Antibiotic prophylaxis should be administered as a single dose from 30 to 60 min before the dental procedure starts. AHA Guidelines [9,10] regimen for IE prophylaxis is reported in Table 6.

In the overall study sample of 242 Italian dentists, knowledge regarding clinical conditions in which antibiotic prophylaxis for infective endocarditis is indicated appeared moderate to low. For example, only 40% and 26% of respondents correctly answered the questions related to mitral valve prolapse and cardiac transplantation, respectively. According to current AHA guidelines, antibiotic prophylaxis is not recommended for these conditions [9,10].

Subgroup analyses revealed heterogeneous patterns in knowledge according to professional experience, academic background, and clinical practice. Overall, no consistent trend was observed linking greater clinical experience with improved knowledge, as both more and less experienced dentists demonstrated strengths and gaps depending on the specific clinical condition. Notably, knowledge of conditions for which antibiotic prophylaxis is clearly recommended by current guidelines, such as previous infective endocarditis, remained suboptimal across all experience levels. Conversely, conditions not requiring prophylaxis, such as implantable cardiac devices or vascular grafts, were frequently misinterpreted, particularly by less experienced practitioners. These findings suggest a partial and inconsistent understanding of guideline indications rather than a simple effect of clinical experience.

Differences according to academic background and clinical practice were also limited. Although pediatric dentistry specialists showed slightly better performance in some areas, incorrect responses remained common even within this group. Similarly, dentists primarily treating adult patients demonstrated higher accuracy for certain conditions; however, overall knowledge gaps persisted. Taken together, these results indicate a generalized uncertainty in identifying clinical conditions requiring antibiotic prophylaxis, regardless of professional profile. This observation is consistent with previous studies reporting variability in guideline adherence among dental practitioners and highlights the need for targeted educational interventions.

Regarding dental procedures for which antibiotic prophylaxis for infective endocarditis is recommended in at-risk patients, the overall level of knowledge in the study sample appeared generally adequate.

In subgroup analyses based on professional experience, a statistically significant difference was observed for procedures involving manipulation of the periapical region of the teeth, with higher accuracy among dentists with less than 10 years and more than 30 years of clinical experience. Similarly, in the subgroup based on the mainly treated population, higher accuracy was found for procedures involving perforation of the oral mucosa among dentists primarily treating pediatric patients. Pediatric dentistry specialists also showed higher correct response rates, although these differences were not statistically significant. In the subgroup based on the sector of professional practice, dentists working in public or mixed (public and private) settings demonstrated significantly higher accuracy for procedures involving manipulation of the periapical region of the teeth, for which antibiotic prophylaxis is recommended according to AHA guidelines. Statistical significance also emerged for “Suture removal”, with a higher correct response rate between dentists who practice the profession only in the private sector; however, most of them answered incorrectly to this question.

Regarding dental procedures requiring antibiotic prophylaxis for infective endocarditis, the overall level of knowledge appeared relatively higher than that observed for clinical conditions. Most respondents correctly identified invasive procedures involving gingival or periapical tissues as indications for prophylaxis, in line with current guidelines. However, subgroup analyses revealed inconsistent patterns. Differences related to professional experience, type of practice, and patient population were observed, but without a clear or consistent trend. For example, higher accuracy was found among both less and more experienced dentists for certain procedures, while practitioners primarily treating pediatric patients demonstrated better knowledge of procedures involving oral mucosal perforation. Similarly, dentists working in public or mixed settings showed higher accuracy for some indications.

Despite these differences, relevant knowledge gaps persisted. In particular, procedures such as suture removal were frequently misinterpreted, even among subgroups with otherwise higher performance. These findings suggest that, although dentists are generally more confident in identifying procedure-related indications, uncertainties remain in borderline or less clearly defined situations. Most respondents correctly identified amoxicillin as the first-line antibiotic for infective endocarditis prophylaxis in pediatric patients without penicillin allergy, in agreement with current guidelines. However, important gaps emerged regarding dosage and timing of administration. Only about half of the participants correctly indicated the recommended dosage, while a substantial proportion reported lower doses, potentially reducing the effectiveness of prophylaxis. Similarly, although the majority correctly identified the recommended timing of administration, a considerable percentage of dentists reported inappropriate regimens, such as split dosing before and after the procedure, which is not supported by current guidelines. These findings highlight a critical discrepancy between general knowledge of the recommended antibiotic and the correct application of its posology. This is clinically relevant, as inappropriate dosing or timing may compromise the effectiveness of prophylaxis and contribute to suboptimal patient management. Although no statistically significant differences were observed among subgroups, pediatric dentistry specialists showed a trend toward better knowledge of the correct dosage. Nevertheless, knowledge gaps persisted across all professional categories, suggesting the need for improved dissemination of practical aspects of guideline recommendations, particularly regarding dosage and administration protocols.

Within the overall sample, macrolides (azithromycin or clarithromycin) were most frequently selected for the prophylaxis of infective endocarditis in pediatric patients with penicillin allergy, being indicated by 48% of respondents. Seventeen percent were unsure, while 28% reported clindamycin, whose use is no longer recommended by the most recent AHA guidelines due to its association with significant adverse reactions, including community-acquired *Clostridium difficile* infections, which may lead to severe complications or death, as reported in a recent study conducted in the United Kingdom. Moreover, up to 15% of community-acquired *Clostridium difficile* infections have been linked to antibiotics prescribed for dental procedures [4,9,10,11]. Only 25% of participants reported the correct dosage of antibiotic prophylaxis according to AHA guidelines based on the selected drug. While 68% correctly identified the recommended timing of administration, 25% indicated inappropriate regimens, such as a single dose administered after the procedure or a split-dose schedule.

In patients with penicillin allergy, no statistically significant differences were observed among subgroups based on academic level, patient population, or primary practice setting. However, when stratified by professional experience, a statistically significant difference was identified in the choice of first-line antibiotic: it was found that dentists who have practiced the profession for more than 30 years are those who would mostly choose the Cephalosporin (14%), despite the preference for the Macrolide. Furthermore, the rate of dentists who have been practicing the profession for more than 30 years who would choose Clindamycin was lower than other experience level groups (14%), in accordance with the AHA Guidelines which no longer recommend this drug. However, dentists with this level of experience are also those who most indicated not knowing the answer (31%) to the question relating to the antibiotic of choice in patients with allergy to penicillins.

In a recent study of Aly and Elchaghaby [12], it was found that around 70% both of generic dentists and specialists in Pediatric dentistry were aware of the antibiotic prophylaxis Guidelines of IE (the Guidelines used as reference were that of AHA published in 2007 [6]) and were adherents to them. In another study [13], it was reported a rate of awareness of the antibiotic prophylactic regimen of 96% for pediatric dentists (the Guidelines used as reference were that of AHA published in 2007 [6] and of the British Society for Antimicrobial Chemotherapy published in 2006 [14]). Although at first glance, the rates of adherence to the guidelines for antibiotic prophylaxis of IE in pediatric dentistry could appear better in the above-mentioned studies if compared to the present one, the questionnaires used by the researchers and the questions asked were different, so it appears difficult to perform a clear comparison.

Surely, the authors of the present study expected greater levels of preparation regarding the antibiotic prophylaxis in pediatric dentistry, particularly between dentist specialists in Pediatric dentistry and who mainly treat pediatric patients (i.e., children and adolescents) in their professional activity. This is because clear and constantly updated Guidelines are published by the AHA. Furthermore, in Europe the Guidelines periodically published by the European Society of Cardiology [15] are available too (the most recent have been published in 2023). These findings highlight the need for improved knowledge of current guidelines for antibiotic prophylaxis of IE in pediatric dentistry, particularly regarding appropriate dosing and timing of administration. In addition, greater awareness of the adverse effects of clindamycin is warranted, as this drug is no longer recommended by recent guidelines, yet it continues to be prescribed by a substantial proportion of Italian dentists for prophylaxis in penicillin-allergic pediatric patients. The authors also aim to have contributed to improving basic knowledge on the correct use of antibiotic prophylaxis for IE in pediatric dentistry by providing participants with educational material at the end of the questionnaire, as well as guidance on reliable sources for ongoing professional updating.

This study has several limitations. First, the use of a convenience sample may restrict the generalizability of the findings to the broader population of Italian dentists. The relatively low response rate may also introduce response bias, as individuals with a greater interest in antibiotic prescribing could have been more inclined to participate. In addition, the questionnaire was not formally validated, which may affect the reliability of the collected data.

Another limitation is the relatively small proportion of pediatric dentistry specialists included in the sample (approximately 10%). However, this proportion is consistent with the current availability of such specialists in Italy, where postgraduate training programs in pediatric dentistry have been introduced only recently and are currently offered by a limited number of universities. Furthermore, the length and complexity of the questionnaire may have discouraged some participants from completing it, potentially leading to dropouts. Conversely, the provision of educational material upon completion may have motivated respondents to complete the survey.

Future research should consider repeating the study as the number of pediatric dentistry specialists increases in Italy and expanding the sample size through larger-scale or multicenter studies involving collaboration among multiple academic institutions.

## 4. Materials and Methods

### 4.1. Questionnaire and Study Sample

A structured questionnaire was developed using the Google Forms platform (Google LLC, Mountain View, CA, USA) and electronically distributed to a group of Italian dentists. The questionnaire was distributed through a mailing list of 700 dentists. A total of 242 completed responses were collected, corresponding to a response rate of 34.6%, calculated based on the number of dentists included in the mailing list. However, it cannot be excluded that the questionnaire was further shared beyond the original mailing list, and therefore the response rate should be interpreted as an estimate.

The questionnaire was developed by the research team based on current international guidelines on antibiotic use in pediatric dentistry and infective endocarditis prophylaxis. Before dissemination, the questionnaire underwent an informal assessment by colleagues with expertise in pediatric dentistry antibiotic prescribing, and current clinical guidelines to evaluate its clarity and content relevance. No formal validation procedures, such as pilot testing, internal consistency assessment, or test–retest reliability analysis, were performed. Participants were asked to provide consent for the use of their anonymized responses for research purposes. The authors also prepared educational material on antibiotic prescribing in pediatric dentistry, which was provided to participants after completion of the questionnaire.

The questionnaire consisted of 3 parts:

1. Demographic information—year of birth, gender, professional experience (i.e., years of dental practice), academic qualification, primary patient population, and practice setting.
2. General knowledge on the prescription of antibiotics in pediatric dentistry—clinical conditions in which the prescription of antibiotics is indicated and practices of use.
3. Prophylaxis of infective endocarditis in pediatric dentistry—anamnestic conditions and dental procedures in which the antibiotic prophylaxis is recommended and practices of prescription.

The American Academy of Pediatric Dentistry (AAPD) guidelines [4,11], the European Academy of Pediatric Dentistry (EAPD) guidelines [16], the AWaRe Antibiotics Manual of the Agenzia Italiana del Farmaco (AIFA) [17], and the American Heart Association (AHA) guidelines [9,10], were used as reference standards to determine the correctness of the responses.

### 4.2. Data Collection

The questionnaire was electronically transmitted to a cohort of Italian dentists. The survey was configured to allow only one response per account through the Google Forms settings, in order to reduce the risk of duplicate submissions.

A convenience sampling strategy was adopted, and participation was voluntary. Therefore, the sample may not be fully representative of the entire population of Italian dentists. The application automatically stopped the completion of the questionnaire if the participant had not provided the consent to research purposes. All questions’ answers were set as mandatory, so the application automatically prevented to continue to complete the questionnaire in case of an answer not provided. Collected data were organized in a table (Excel, Microsoft Office 365, Microsoft, Remond, WA, USA).

### 4.3. Statistical Analysis

Statistical analyses were conducted by the Department of Information Engineering at the University of Padova (Italy). Categorical variables were summarized as frequencies and percentages, while continuous variables were reported as medians and interquartile ranges (IQR). Group comparisons for categorical data were performed using the chi-square test or Fisher’s exact test, as appropriate. Specifically, Fisher’s exact test was applied when the assumptions for the chi-square test were not met, particularly in the presence of expected cell counts below 5.

No adjustment for multiple comparisons was performed, as the study was exploratory in nature. All tests were two-tailed, and a *p*-value < 0.05 was considered statistically significant. Statistical analyses were carried out using R version 4.3 (R Foundation for Statistical Computing, Vienna, Austria; R Core Team, 2023. R: A Language and Environment for Statistical Computing).

## 5. Conclusions

Within the limitations of this study, the results highlight important gaps in the knowledge and prescribing practices of Italian dentists regarding antibiotic prophylaxis for infective endocarditis in pediatric patients. Although the first-line antibiotic is generally correctly identified, significant uncertainties persist regarding its dosage, timing of administration, and the choice of alternative antibiotics. These findings underline the need for improved dissemination and implementation of current guidelines to promote appropriate antibiotic use and support antibiotic stewardship in dental practice.

## Figures and Tables

**Table 1:** Distribution of the sample based on demographic characteristics.

Variable	Category	All (*n* = 242)
Sex	Woman	164 (68%)
	I prefer not to answer	4 (2%)
	Man	74 (31%)
Years of dental practice	10 years or less	92 (38%)
	11–20 years	75 (31%)
	21–30 years	39 (16%)
	More than 30 years	36 (15%)
Academic level	Oral surgery specialist	5 (2%)
	Pediatric dentistry specialist	24 (10%)
	Orthodontics specialist	73 (30%)
	Generic dentist	140 (58%)
Mainly treated population	Adults	32 (13%)
	Adults and children/teenagers	138 (57%)
	Children/teenagers	72 (30%)
Sector of dental profession practicing	Private	187 (77%)
	Public	10 (4%)
	Public and private	45 (19%)
Geographic region	North Italy	170 (70%)
	Central Italy	48 (20%)
	South Italy and Islands	24 (10%)

**Table 2:** Right/wrong responses for clinical conditions in which antibiotic prophylaxis of IE is recommended in the total sample.

Variable	Category	All (*n* = 242)
Mitral valve prolapse	No	96 (40%)
	I don’t know	14 (6%)
	Yes	132 (55%)
Prosthetic cardiac valves, including transcatheter-implanted prostheses and homografts	No	8 (3%)
	I don’t know	4 (2%)
	Yes	230 (95%)
Septal defect closure devices when complete closure is achieved	No	124 (51%)
	I don’t know	37 (15%)
	Yes	81 (33%)
Prosthetic material used for cardiac valve repair	No	23 (10%)
	I don’t know	16 (7%)
	Yes	203 (84%)
Previous IE	No	17 (7%)
	I don’t know	8 (3%)
	Yes	217 (90%)
Unrepaired cyanotic congenital heart defect (CHD)	No	54 (22%)
	I don’t know	32 (13%)
	Yes	156 (64%)
Repaired CHD, with residual shunts or valvular regurgitation at the site of or adjacent to the site of a prosthetic patch or prosthetic device	No	17 (7%)
	I don’t know	33 (14%)
	Yes	192 (79%)
Implantable electronic devices such as a pacemaker or similar devices	No	167 (69%)
	I don’t know	20 (8%)
	Yes	55 (23%)
Cardiac transplant	No	63 (26%)
	I don’t know	33 (14%)
	Yes	146 (60%)
Cardiac transplant with valve regurgitation due to a structurally abnormal valve	No	5 (2%)
	I don’t know	22 (9%)
	Yes	215 (89%)
Peripheral vascular grafts and patches, including those used for hemodialysis	No	88 (36%)
	I don’t know	51 (21%)
	Yes	103 (43%)
Coronary artery stents or other vascular stents	No	120 (50%)
	I don’t know	36 (15%)
	Yes	86 (36%)

**Table 3:** Right/wrong responses for dental procedures in which antibiotic prophylaxis of IE is recommended in the total sample.

Variable	Category	All (*n* = 242)
Routine anesthetic injections through noninfected tissue	No	218 (90%)
	I don’t know	3 (1%)
	Yes	21 (9%)
Procedures that involve manipulation of the gingival tissue	No	56 (23%)
	I don’t know	8 (3%)
	Yes	178 (74%)
Bleeding from trauma to the lips or oral mucosa	No	114 (47%)
	I don’t know	13 (5%)
	Yes	115 (48%)
Procedures that involve manipulation of the periapical region of teeth	No	86 (36%)
	I don’t know	14 (6%)
	Yes	142 (59%)
Procedures that involve perforation of the oral mucosa	No	59 (24%)
	I don’t know	11 (5%)
	Yes	172 (71%)
Shedding of deciduous teeth	No	240 (99%)
	I don’t know	1 (0%)
	Yes	1 (0%)
Placement of removable prosthodontic or orthodontic appliances	No	239 (99%)
	I don’t know	1 (0%)
	Yes	2 (1%)
Adjustment of orthodontic appliances	No	242 (100%)
Placement of orthodontic brackets	No	242 (100%)
Taking dental radiographs	No	241 (100%)
	I don’t know	1 (0%)
Suture removal	No	221 (91%)
	I don’t know	7 (3%)
	Yes	14 (6%)

**Table 4:** Knowledge on the antibiotic of first choice for the prophylaxis of IE in patients with no allergy to penicillins in the total sample.

Variable	Category	All (*n* = 242)
Antibiotic of first choice for the prophylaxis of IE in pediatric patient with no allergy to penicillins able to take oral medication	Amoxicillin	226 (93%)
	Azithromycin or clarithromycin	1 (0%)
	Cefazolin or ceftriaxone	2 (1%)
	I don’t know	13 (5%)
Dosage (Amoxicillin)	Low	89 (39%)
	Right	112 (50%)
	I don’t know	25 (11%)
Timing (Amoxicillin)	Double dose: half 30–60 min before the procedure + half 30–60 min after the procedure	58 (26%)
	I don’t know	9 (4%)
	Single dose 30–60 min after procedure	6 (3%)
	Single dose 30–60 min before procedure	153 (68%)

**Table 5:** Knowledge on the antibiotic of first choice for the prophylaxis of IE in patients with allergy to penicillins in the total sample.

Variable	Category	All (*n* = 242)
Antibiotic of first choice for the prophylaxis of IE in pediatric patient with allergy to penicillins able to take oral medication	Amoxicillin	2 (1%)
	Azithromycin or clarithromycin	117 (48%)
	Cephalosporin	14 (6%)
	Cefazolin or ceftriaxone	1 (0%)
	Clindamycin	67 (28%)
	I don’t know	41 (17%)
Dosage (correct antibiotic)	High	26 (13%)
	Low	81 (41%)
	Right	49 (25%)
	I don’t know	42 (21%)
Timing (correct antibiotic)	Double dose: half 30–60 min before the procedure + half 30–60 min after the procedure	35 (18%)
	I don’t know	16 (8%)
	Single dose 30–60 min after procedure	13 (7%)
	Single dose 30–60 min before procedure	134 (68%)

**Table 6:** AHA Guidelines (2021) [9,10] antibiotic prophylaxis regimen for dental procedures in the pediatric patient at risk for IE.

Situation	Agent	Children
Oral	Amoxicillin	50 mg/kg
Unable to take oral medication	Ampicillin	50 mg/kg IM o IV
	or	
	Cefazolin or ceftriaxone	50 mg/kg IM o IV
Allergic to penicillins or ampicillin—oral regimen	Cephalexin	50 mg/kg
	or	
	Azithromycin or clarithromycin	15 mg/kg
	or	
	Doxycycline	<45 kg, 2.2 mg/kg >45 kg, 100 mg
Allergic to penicillin or ampicillin and unable to take oral medication	Cefazolin or ceftriaxone	50 mg/kg IM o IV

## Data Availability

Data are available upon request made to the corresponding author.

## Supplementary Materials

The following supporting information can be downloaded at: https://www.mdpi.com/article/10.3390/antibiotics15050460/s1.
