# Supplementary material for: Antibiotics for Prophylaxis of Infective Endocarditis in Pediatric Patients: Knowledge and Prescribing Practices Between Italian Dentists
Source: Antibiotics (Basel). 2026 May 1;15(5):460. doi: 10.3390/antibiotics15050460 (PMC13203871; doi:10.3390/antibiotics15050460)
Supplement: Supplementary file 1 [file antibiotics-15-00460-s001.zip › antibiotics-4172763-supplementary.pdf]

SUPPLEMENTARY MATERIALS

Table S1. Right/wrong responses for clinical conditions in which antibiotic prophylaxis of IE is recommended on the basis of experience (years of dental practice).

Variable	Category	10 years or less (n=92)	11-20 years (n=75)	21-30 years (n=39)	More than 30 years (n=36)	p-value
Mitral valve prolapse	No	40 (43%)	32 (43%)	19 (49%)	5 (14%)	0.005
	I don't know	4 (4%)	5 (7%)	0 (0%)	5 (14%)	
	Yes	48 (52%)	38 (51%)	20 (51%)	26 (72%)	
Prosthetic cardiac valves, including transcatheter-implanted prostheses and homografts	No	3 (3%)	2 (3%)	1 (3%)	2 (6%)	0.94
	I don't know	2 (2%)	2 (3%)	0 (0%)	0 (0%)	
	Yes	87 (95%)	71 (95%)	38 (97%)	34 (94%)	
Septal defect closure devices when complete closure is achieved	No	50 (54%)	44 (59%)	15 (38%)	15 (42%)	0.11
	I don't know	9 (10%)	14 (19%)	7 (18%)	7 (19%)	
	Yes	33 (36%)	17 (23%)	17 (44%)	14 (39%)	
Prosthetic material used for cardiac valve repair	No	8 (9%)	6 (8%)	4 (10%)	5 (14%)	0.12
	I don't know	3 (3%)	3 (4%)	6 (15%)	4 (11%)	
	Yes	81 (88%)	66 (88%)	29 (74%)	27 (75%)	
Previous IE	No	2 (2%)	3 (4%)	8 (21%)	4 (11%)	0.003
	I don't know	2 (2%)	5 (7%)	1 (3%)	0 (0%)	
	Yes	88 (96%)	67 (89%)	30 (77%)	32 (89%)	
Unrepaired cyanotic congenital heart defect (CHD)	No	30 (33%)	14 (19%)	8 (21%)	2 (6%)	0.02
	I don't know	9 (10%)	13 (17%)	6 (15%)	4 (11%)	
	Yes	53 (58%)	48 (64%)	25 (64%)	30 (83%)	
Repaired CHD, with residual shunts or valvular regurgitation at the site of or adjacent to the site of a prosthetic patch or prosthetic device	No	7 (8%)	7 (9%)	3 (8%)	0 (0%)	0.24
	I don't know	8 (9%)	11 (15%)	8 (21%)	6 (17%)	
	Yes	77 (84%)	57 (76%)	28 (72%)	30 (83%)	
Implantable electronic devices such as a pacemaker or similar devices	No	70 (76%)	45 (60%)	27 (69%)	25 (69%)	0.04
	I don't know	2 (2%)	8 (11%)	6 (15%)	4 (11%)	
	Yes	20 (22%)	22 (29%)	6 (15%)	7 (19%)	
Cardiac transplant	No	30 (33%)	20 (27%)	10 (26%)	3 (8%)	0.08
	I don't know	14 (15%)	9 (12%)	3 (8%)	7 (19%)	
	Yes	48 (52%)	46 (61%)	26 (67%)	26 (72%)	
Cardiac transplant with valve regurgitation due to a structurally abnormal valve	No	3 (3%)	1 (1%)	1 (3%)	0 (0%)	0.84
	I don't know	9 (10%)	5 (7%)	5 (13%)	3 (8%)	
	Yes	80 (87%)	69 (92%)	33 (85%)	33 (92%)	

Peripheral vascular grafts and patches, including those used for hemodialysis	No	45 (49%)	21 (28%)	13 (33%)	9 (25%)	0.01
	I don't know	15 (16%)	13 (17%)	12 (31%)	11 (31%)	
	Yes	32 (35%)	41 (55%)	14 (36%)	16 (44%)	
Coronary artery stents or other vascular stents	No	56 (61%)	38 (51%)	15 (38%)	11 (31%)	0.04
	I don't know	9 (10%)	11 (15%)	9 (23%)	7 (19%)	
	Yes	27 (29%)	26 (35%)	15 (38%)	18 (50%)	

Table S2. Right/wrong responses for clinical conditions in which antibiotic prophylaxis of IE is recommended on the basis of academic level.

Variable	Category	General dentist (n=141)	Orthodontics or Oral surgery specialist (n=77)	Pediatric dentistry specialist (n=24)	p-value
Mitral valve prolapse	No	57 (40%)	31 (40%)	8 (33%)	0.34
	I don't know	5 (4%)	6 (8%)	3 (13%)	
	Yes	79 (56%)	40 (52%)	13 (54%)	
Prosthetic cardiac valves, including transcatheter-implanted prostheses and homografts	No	1 (1%)	5 (6%)	2 (8%)	0.05
	I don't know	3 (2%)	1 (1%)	0 (0%)	
	Yes	137 (97%)	71 (92%)	22 (92%)	
Septal defect closure devices when complete closure is achieved	No	72 (51%)	39 (51%)	13 (54%)	0.62
	I don't know	18 (13%)	14 (18%)	5 (21%)	
	Yes	51 (36%)	24 (31%)	6 (25%)	
Prosthetic material used for cardiac valve repair	No	12 (9%)	8 (10%)	3 (13%)	0.59
	I don't know	9 (6%)	7 (9%)	0 (0%)	
	Yes	120 (85%)	62 (81%)	21 (88%)	
Previous IE	No	10 (7%)	7 (9%)	0 (0%)	0.36
	I don't know	3 (2%)	4 (5%)	1 (4%)	
	Yes	128 (91%)	66 (86%)	23 (96%)	
Unrepaired cyanotic congenital heart defect (CHD)	No	28 (20%)	20 (26%)	6 (25%)	0.82
	I don't know	20 (14%)	10 (13%)	2 (8%)	
	Yes	93 (66%)	47 (61%)	16 (67%)	
Repaired CHD, with residual shunts or valvular regurgitation at the site of or adjacent to the site of a prosthetic patch or prosthetic device	No	6 (4%)	8 (10%)	3 (13%)	0.17
	I don't know	21 (15%)	11 (14%)	1 (4%)	
	Yes	114 (81%)	58 (75%)	20 (83%)	
Implantable electronic devices such as a pacemaker or similar devices	No	98 (70%)	49 (64%)	20 (83%)	0.38
	I don't know	13 (9%)	7 (9%)	0 (0%)	
	Yes	30 (21%)	21 (27%)	4 (17%)	
Cardiac transplant	No	36 (26%)	17 (22%)	10 (42%)	0.02

	I don't know	16 (11%)	17 (22%)	0 (0%)	
	Yes	89 (63%)	43 (56%)	14 (58%)	
Cardiac transplant with valve regurgitation due to a structurally abnormal valve	No	2 (1%)	2 (3%)	1 (4%)	0.22
	I don't know	10 (7%)	11 (14%)	1 (4%)	
	Yes	129 (91%)	64 (83%)	22 (92%)	
Peripheral vascular grafts and patches, including those used for hemodialysis	No	51 (36%)	21 (27%)	16 (67%)	0.01
	I don't know	28 (20%)	21 (27%)	2 (8%)	
	Yes	62 (44%)	35 (45%)	6 (25%)	
Coronary artery stents or other vascular stents	No	75 (53%)	30 (39%)	15 (63%)	0.16
	I don't know	18 (13%)	16 (21%)	2 (8%)	
	Yes	48 (34%)	31 (40%)	7 (29%)	

Table S3. Right/wrong responses for clinical conditions in which antibiotic prophylaxis of IE is recommended on the basis of mainly treated population.

Variable	Category	Adults (n=32)	Adults and children/teenagers (n=138)	Children/teenagers (n=72)	p-value
Mitral valve prolapse	No	18 (56%)	52 (38%)	26 (36%)	0.21
	I don't know	0 (0%)	8 (6%)	6 (8%)	
	Yes	14 (44%)	78 (57%)	40 (56%)	
Prosthetic cardiac valves, including transcatheter-implanted prostheses and homografts	No	0 (0%)	4 (3%)	4 (6%)	0.73
	I don't know	0 (0%)	3 (2%)	1 (1%)	
	Yes	32 (100%)	131 (95%)	67 (93%)	
Septal defect closure devices when complete closure is achieved	No	15 (47%)	72 (52%)	37 (51%)	0.23
	I don't know	2 (6%)	20 (14%)	15 (21%)	
	Yes	15 (47%)	46 (33%)	20 (28%)	
Prosthetic material used for cardiac valve repair	No	3 (9%)	17 (12%)	3 (4%)	0.35
	I don't know	1 (3%)	9 (7%)	6 (8%)	
	Yes	28 (88%)	112 (81%)	63 (88%)	
Previous IE	No	3 (9%)	13 (9%)	1 (1%)	0.15
	I don't know	1 (3%)	4 (3%)	3 (4%)	
	Yes	28 (88%)	121 (88%)	68 (94%)	
Unrepaired cyanotic congenital heart defect (CHD)	No	5 (16%)	34 (25%)	15 (21%)	0.46
	I don't know	2 (6%)	18 (13%)	12 (17%)	
	Yes	25 (78%)	86 (62%)	45 (63%)	
Repaired CHD, with residual shunts or valvular regurgitation at the site of or adjacent to the site of a	No	0 (0%)	13 (9%)	4 (6%)	0.30
	I don't know	3 (9%)	18 (13%)	12 (17%)	
	Yes	29 (91%)	107 (78%)	56 (78%)	

prosthetic patch or prosthetic device					
Implantable electronic devices such as a pacemaker or similar devices	No	21 (66%)	98 (71%)	48 (67%)	0.84
	I don't know	3 (9%)	12 (9%)	5 (7%)	
	Yes	8 (25%)	28 (20%)	19 (26%)	
Cardiac transplant	No	14 (44%)	33 (24%)	16 (22%)	0.02
	I don't know	0 (0%)	22 (16%)	11 (15%)	
	Yes	18 (56%)	83 (60%)	45 (63%)	
Cardiac transplant with valve regurgitation due to a structurally abnormal valve	No	1 (3%)	4 (3%)	0 (0%)	0.04
	I don't know	0 (0%)	11 (8%)	11 (15%)	
	Yes	31 (97%)	123 (89%)	61 (85%)	
Peripheral vascular grafts and patches, including those used for hemodialysis	No	16 (50%)	46 (33%)	26 (36%)	0.14
	I don't know	2 (6%)	31 (22%)	18 (25%)	
	Yes	14 (44%)	61 (44%)	28 (39%)	
Coronary artery stents or other vascular stents	No	19 (59%)	68 (49%)	33 (46%)	0.20
	I don't know	1 (3%)	20 (14%)	15 (21%)	
	Yes	12 (38%)	50 (36%)	24 (33%)	

Table S4. Right/wrong responses for clinical conditions in which antibiotic prophylaxis of IE is recommended on the basis of sector of dental profession practicing.

Variable	Category	Private (n=187)	Public and public/private (n=55)	p-value
Mitral valve prolapse	No	76 (41%)	20 (36%)	0.18
	I don't know	8 (4%)	6 (11%)	
	Yes	103 (55%)	29 (53%)	
Prosthetic cardiac valves, including transcatheter-implanted prostheses and homografts	No	5 (3%)	3 (5%)	0.52
	I don't know	3 (2%)	1 (2%)	
	Yes	179 (96%)	51 (93%)	
Septal defect closure devices when complete closure is achieved	No	92 (49%)	32 (58%)	0.21
	I don't know	27 (14%)	10 (18%)	
	Yes	68 (36%)	13 (24%)	
Prosthetic material used for cardiac valve repair	No	19 (10%)	4 (7%)	0.61
	I don't know	11 (6%)	5 (9%)	
	Yes	157 (84%)	46 (84%)	
Previous IE	No	15 (8%)	2 (4%)	0.52
	I don't know	7 (4%)	1 (2%)	
	Yes	165 (88%)	52 (95%)	
	No	46 (25%)	8 (15%)	0.27

Unrepaired cyanotic congenital heart defect (CHD)	I don't know	25 (13%)	7 (13%)	
	Yes	116 (62%)	40 (73%)	
Repaired CHD, with residual shunts or valvular regurgitation at the site of or adjacent to the site of a prosthetic patch or prosthetic device	No	15 (8%)	2 (4%)	0.48
	I don't know	24 (13%)	9 (16%)	
	Yes	148 (79%)	44 (80%)	
Implantable electronic devices such as a pacemaker or similar devices	No	129 (69%)	38 (69%)	0.3
	I don't know	13 (7%)	7 (13%)	
	Yes	45 (24%)	10 (18%)	
Cardiac transplant	No	43 (23%)	20 (36%)	0.12
	I don't know	28 (15%)	5 (9%)	
	Yes	116 (62%)	30 (55%)	
Cardiac transplant with valve regurgitation due to a structurally abnormal valve	No	3 (2%)	2 (4%)	0.6
	I don't know	17 (9%)	5 (9%)	
	Yes	167 (89%)	48 (87%)	
Peripheral vascular grafts and patches, including those used for hemodialysis	No	65 (35%)	23 (42%)	0.54
	I don't know	39 (21%)	12 (22%)	
	Yes	83 (44%)	20 (36%)	
Coronary artery stents or other vascular stents	No	91 (49%)	29 (53%)	0.66
	I don't know	30 (16%)	6 (11%)	
	Yes	66 (35%)	20 (36%)	

Table S5. Right/wrong responses for dental procedures in which antibiotic prophylaxis of IE is recommended on the basis of experience (years of dental practice).

Variable	Category	10 years or less (n=92)	11-20 years (n=75)	21-30 years (n=39)	More than 30 years (n=36)	p-value
Routine anesthetic injections through noninfected tissue	No	81 (88%)	69 (92%)	35 (90%)	33 (92%)	0.85
	I don't know	1 (1%)	1 (1%)	0 (0%)	1 (3%)	
	Yes	10 (11%)	5 (7%)	4 (10%)	2 (6%)	
Procedures that involve manipulation of the gingival tissue	No	21 (23%)	17 (23%)	8 (21%)	10 (28%)	0.44
	I don't know	1 (1%)	5 (7%)	2 (5%)	0 (0%)	
	Yes	70 (76%)	53 (71%)	29 (74%)	26 (72%)	
Bleeding from trauma to the lips or oral mucosa	No	44 (48%)	33 (44%)	17 (44%)	20 (56%)	0.12
	I don't know	2 (2%)	3 (4%)	6 (15%)	2 (6%)	
	Yes	46 (50%)	39 (52%)	16 (41%)	14 (39%)	
Procedures that involve manipulation of the periapical region of teeth	No	30 (33%)	31 (41%)	19 (49%)	6 (17%)	0.005
	I don't know	2 (2%)	8 (11%)	1 (3%)	3 (8%)	
	Yes	60 (65%)	36 (48%)	19 (49%)	27 (75%)	
	No	24 (26%)	15 (20%)	12 (31%)	8 (22%)	0.31

Procedures that involve perforation of the oral mucosa	I don't know	1 (1%)	5 (7%)	2 (5%)	3 (8%)	
	Yes	67 (73%)	55 (73%)	25 (64%)	25 (69%)	
Shedding of deciduous teeth	No	91 (99%)	74 (99%)	39 (100%)	36 (100%)	0.85
	I don't know	0 (0%)	1 (1%)	0 (0%)	0 (0%)	
	Yes	1 (1%)	0 (0%)	0 (0%)	0 (0%)	
Placement of removable prosthodontic or orthodontic appliances	No	92 (100%)	73 (97%)	39 (100%)	35 (97%)	0.08
	I don't know	0 (0%)	0 (0%)	0 (0%)	1 (3%)	
	Yes	0 (0%)	2 (3%)	0 (0%)	0 (0%)	
Adjustment of orthodontic appliances	No	92 (100%)	75 (100%)	39 (100%)	36 (100%)	NA
Placement of orthodontic brackets	No	92 (100%)	75 (100%)	39 (100%)	36 (100%)	NA
Taking dental radiographs	No	92 (100%)	75 (100%)	39 (100%)	35 (97%)	0.14
	I don't know	0 (0%)	0 (0%)	0 (0%)	1 (3%)	
Suture removal	No	80 (87%)	73 (97%)	36 (92%)	32 (89%)	0.21
	I don't know	3 (3%)	1 (1%)	1 (3%)	2 (6%)	
	Yes	9 (10%)	1 (1%)	2 (5%)	2 (6%)	

Table S6. Right/wrong responses for dental procedures in which antibiotic prophylaxis of IE is recommended on the basis of academic level.

Variable	Category	General dentist (n=141)	Orthodontics or Oral surgery specialist (n=77)	Pediatric dentistry specialist (n=24)	p-value
Routine anesthetic injections through noninfected tissue	No	130 (92%)	69 (90%)	19 (79%)	0.21
	I don't know	1 (1%)	1 (1%)	1 (4%)	
	Yes	10 (7%)	7 (9%)	4 (17%)	
Procedures that involve manipulation of the gingival tissue	No	31 (22%)	21 (27%)	4 (17%)	0.28
	I don't know	3 (2%)	5 (6%)	0 (0%)	
	Yes	107 (76%)	51 (66%)	20 (83%)	
Bleeding from trauma to the lips or oral mucosa	No	72 (51%)	32 (42%)	10 (42%)	0.34
	I don't know	5 (4%)	7 (9%)	1 (4%)	
	Yes	64 (45%)	38 (49%)	13 (54%)	
Procedures that involve manipulation of the periapical region of teeth	No	48 (34%)	33 (43%)	5 (21%)	0.12
	I don't know	8 (6%)	6 (8%)	0 (0%)	
	Yes	85 (60%)	38 (49%)	19 (79%)	
Procedures that involve perforation of the oral mucosa	No	39 (28%)	18 (23%)	2 (8%)	0.17
	I don't know	8 (6%)	3 (4%)	0 (0%)	
	Yes	94 (67%)	56 (73%)	22 (92%)	
Shedding of deciduous teeth	No	140 (99%)	76 (99%)	24 (100%)	0.66

	I don't know	0 (0%)	1 (1%)	0 (0%)	
	Yes	1 (1%)	0 (0%)	0 (0%)	
Placement of removable prosthodontic or orthodontic appliances	No	139 (99%)	76 (99%)	24 (100%)	0.99
	I don't know	1 (1%)	0 (0%)	0 (0%)	
	Yes	1 (1%)	1 (1%)	0 (0%)	
Adjustment of orthodontic appliances	No	141 (100%)	77 (100%)	24 (100%)	NA
Placement of orthodontic brackets	No	141 (100%)	77 (100%)	24 (100%)	NA
Taking dental radiographs	No	141 (100%)	76 (99%)	24 (100%)	0.41
	I don't know	0 (0%)	1 (1%)	0 (0%)	
Suture removal	No	129 (91%)	72 (94%)	20 (83%)	0.34
	I don't know	3 (2%)	2 (3%)	2 (8%)	
	Yes	9 (6%)	3 (4%)	2 (8%)	

Table S7. Right/wrong responses for dental procedures in which antibiotic prophylaxis of IE is recommended on the basis of mainly treated population.

Variable	Category	Adults (n=32)	Adults and children/teenagers (n=138)	Children/teenagers (n=72)	p-value
Routine anesthetic injections through noninfected tissue	No	32 (100%)	123 (89%)	63 (88%)	0.19
	I don't know	0 (0%)	1 (1%)	2 (3%)	
	Yes	0 (0%)	14 (10%)	7 (10%)	
Procedures that involve manipulation of the gingival tissue	No	9 (28%)	35 (25%)	12 (17%)	0.48
	I don't know	0 (0%)	5 (4%)	3 (4%)	
	Yes	23 (72%)	98 (71%)	57 (79%)	
Bleeding from trauma to the lips or oral mucosa	No	18 (56%)	66 (48%)	30 (42%)	0.18
	I don't know	0 (0%)	11 (8%)	2 (3%)	
	Yes	14 (44%)	61 (44%)	40 (56%)	
Procedures that involve manipulation of the periapical region of teeth	No	16 (50%)	51 (37%)	19 (26%)	0.21
	I don't know	1 (3%)	8 (6%)	5 (7%)	
	Yes	15 (47%)	79 (57%)	48 (67%)	
Procedures that involve perforation of the oral mucosa	No	12 (38%)	39 (28%)	8 (11%)	0.005
	I don't know	0 (0%)	5 (4%)	6 (8%)	
	Yes	20 (63%)	94 (68%)	58 (81%)	
Shedding of deciduous teeth	No	32 (100%)	138 (100%)	70 (97%)	0.33
	I don't know	0 (0%)	0 (0%)	1 (1%)	
	Yes	0 (0%)	0 (0%)	1 (1%)	
	No	32 (100%)	135 (98%)	72 (100%)	0.8

Placement of removable prosthodontic or orthodontic appliances	I don't know	0 (0%)	1 (1%)	0 (0%)	
	Yes	0 (0%)	2 (1%)	0 (0%)	
Adjustment of orthodontic appliances	No	32 (100%)	138 (100%)	72 (100%)	NA
Placement of orthodontic brackets	No	32 (100%)	138 (100%)	72 (100%)	NA
Taking dental radiographs	No	32 (100%)	138 (100%)	71 (99%)	0.42
	I don't know	0 (0%)	0 (0%)	1 (1%)	
Suture removal	No	31 (97%)	124 (90%)	66 (92%)	0.77
	I don't know	0 (0%)	4 (3%)	3 (4%)	
	Yes	1 (3%)	10 (7%)	3 (4%)	

Table S8. Right/wrong responses for dental procedures in which antibiotic prophylaxis of IE is recommended on the basis of sector of dental profession practicing.

Variable	Category	Private (n=187)	Public and public/private (n=55)	p-value
Routine anesthetic injections through noninfected tissue	No	171 (91%)	47 (85%)	0.11
	I don't know	1 (1%)	2 (4%)	
	Yes	15 (8%)	6 (11%)	
Procedures that involve manipulation of the gingival tissue	No	44 (24%)	12 (22%)	0.58
	I don't know	5 (3%)	3 (5%)	
	Yes	138 (74%)	40 (73%)	
Bleeding from trauma to the lips or oral mucosa	No	90 (48%)	24 (44%)	0.35
	I don't know	8 (4%)	5 (9%)	
	Yes	89 (48%)	26 (47%)	
Procedures that involve manipulation of the periapical region of teeth	No	76 (41%)	10 (18%)	0.002
	I don't know	8 (4%)	6 (11%)	
	Yes	103 (55%)	39 (71%)	
Procedures that involve perforation of the oral mucosa	No	47 (25%)	12 (22%)	0.88
	I don't know	9 (5%)	2 (4%)	
	Yes	131 (70%)	41 (75%)	
Shedding of deciduous teeth	No	185 (99%)	55 (100%)	0.99
	I don't know	1 (1%)	0 (0%)	
	Yes	1 (1%)	0 (0%)	
Placement of removable prosthodontic or orthodontic appliances	No	186 (99%)	53 (96%)	0.13
	I don't know	0 (0%)	1 (2%)	
	Yes	1 (1%)	1 (2%)	
Adjustment of orthodontic appliances	No	187 (100%)	55 (100%)	NA

Placement of orthodontic brackets	No	187 (100%)	55 (100%)	NA
Taking dental radiographs	No	186 (99%)	55 (100%)	0.99
	I don't know	1 (1%)	0 (0%)	
Suture removal	No	175 (94%)	46 (84%)	0.03
	I don't know	3 (2%)	4 (7%)	
	Yes	9 (5%)	5 (9%)	

Table S9. Knowledge on the antibiotic of first choice for the prophylaxis of IE in patients with no allergy to penicillins on the basis of experience (years of dental practice).

Variable	Category	10 years or less (n=92)	11-20 years (n=75)	21-30 years (n=39)	More than 30 years (n=36)	p-value
Antibiotic of first choice for the prophylaxis of IE in pediatric patient with no allergy to penicillins able to take oral medication	Amoxicillin	86 (93%)	72 (96%)	38 (97%)	30 (83%)	0.13
	Azithromycin or clarithromycin	0 (0%)	1 (1%)	0 (0%)	0 (0%)	
	Cefazolin or ceftriaxone	1 (1%)	0 (0%)	0 (0%)	1 (3%)	
	I don’t know	5 (5%)	2 (3%)	1 (3%)	5 (14%)	
Dosage *(Amoxicillin)*	Low	42 (49%)	22 (31%)	12 (32%)	13 (43%)	0.32
	Right	37 (43%)	40 (56%)	21 (55%)	14 (47%)	
	I don’t know	7 (8%)	10 (14%)	5 (13%)	3 (10%)	
Timing *(Amoxicillin)*	Double dose: half 30-60 minutes before the procedure + half 30-60 minutes after the procedure	25 (29%)	19 (26%)	4 (11%)	10 (33%)	0.31
	I don’t know	5 (6%)	3 (4%)	0 (0%)	1 (3%)	
	Single dose 30-60 minutes after procedure	2 (2%)	2 (3%)	2 (5%)	0 (0%)	
	Single dose 30-60 minutes before procedure	54 (63%)	48 (67%)	32 (84%)	19 (63%)	

Table S10. Knowledge on the antibiotic of first choice for the prophylaxis of IE in patients with no allergy to penicillins on the basis of academic level.

Variable	Category	General dentist (n=141)	Orthodontics or Oral surgery specialist (n=77)	Pediatric dentistry specialist (n=24)	p-value
Antibiotic of first choice for the prophylaxis of IE in pediatric patient	Amoxicillin	132 (94%)	71 (92%)	23 (96%)	0.28
	Azithromycin or	0 (0%)	0 (0%)	1 (4%)	

with no allergy to penicillins able to take oral medication	clarithromycin				
	Cefazolin or ceftriaxone	1 (1%)	1 (1%)	0 (0%)	
	I don't know	8 (6%)	5 (6%)	0 (0%)	
Dosage *(Amoxicillin)*	Low	54 (41%)	28 (39%)	7 (30%)	0.22
	Right	63 (48%)	33 (47%)	16 (70%)	
	I don't know	15 (11%)	10 (14%)	0 (0%)	
Timing *(Amoxicillin)*	Double dose: half 30-60 minutes before the procedure + half 30-60 minutes after the procedure	37 (23%)	17 (24%)	4 (17%)	0.84
	I don't know	4 (3%)	4 (6%)	1 (4%)	
	Single dose 30-60 minutes after procedure	4 (3%)	1 (1%)	1 (4%)	
	Single dose 30-60 minutes before procedure	87 (66%)	49 (69%)	17 (75%)	

Table S11. Knowledge on the antibiotic of first choice for the prophylaxis of IE in patients with no allergy to penicillins on the basis of mainly treated population.

Variable	Category	Adults (n=32)	Adults and children/teenagers (n=138)	Children/teenagers (n=72)	p-value
Antibiotic of first choice for the prophylaxis of IE in pediatric patient with no allergy to penicillins able to take oral medication	Amoxicillin	31 (97%)	129 (93%)	66 (92%)	0.28
	Azithromycin or clarithromycin	0 (0%)	0 (0%)	1 (1%)	
	Cefazolin or ceftriaxone	0 (0%)	0 (0%)	2 (3%)	
	I don't know	1 (3%)	9 (7%)	3 (4%)	
Dosage *(Amoxicillin)*	Low	13 (42%)	50 (39%)	26 (39%)	0.81
	Right	15 (48%)	62 (48%)	35 (53%)	
	I don't know	3 (10%)	17 (13%)	5 (8%)	
Timing *(Amoxicillin)*	Double dose: half 30-60 minutes before the procedure + half 30-60 minutes after the procedure	6 (19%)	34 (24%)	18 (23%)	0.77

	I don't know	0 (0%)	5 (6%)	4 (6%)	
	Single dose 30-60 minutes after procedure	1 (3%)	3 (2%)	2 (3%)	
	Single dose 30-60 minutes before procedure	24 (77%)	87 (67%)	42 (64%)	

Table S12. Knowledge on the antibiotic of first choice for the prophylaxis of IE in patients with no allergy to penicillins on the basis of sector of dental profession practicing.

Variable	Category	Private (n=187)	Public and public/private (n=55)	p-value
Antibiotic of first choice for the prophylaxis of IE in pediatric patient with no allergy to penicillins able to take oral medication	Amoxicillin	176 (94%)	50 (91%)	0.43
	Azithromycin or clarithromycin	1 (1%)	0 (0%)	
	Cefazolin or ceftriaxone	2 (1%)	0 (0%)	
	I don't know	8 (4%)	5 (9%)	
Dosage *(Amoxicillin)*	Low	68 (38%)	21 (42%)	0.32
	Right	91 (52%)	21 (42%)	
	I don't know	17 (10%)	8 (16%)	
Timing *(Amoxicillin)*	Double dose: half 30-60 minutes before the procedure + half 30-60 minutes after the procedure	43 (24%)	15 (30%)	0.53
	I don't know	7 (4%)	2 (4%)	
	Single dose 30-60 minutes after procedure	6 (3%)	0 (0%)	
	Single dose 30-60 minutes before procedure	120 (68%)	33 (66%)	

Table S13. Knowledge on the antibiotic of first choice for the prophylaxis of IE in patients with no allergy to penicillins on the basis of experience (years of dental practice).

Variable	Category	10 years or less (n=92)	11-20 years (n=75)	21-30 years (n=39)	More than 30 years (n=36)	p-value
Antibiotic of first choice for the prophylaxis of IE in pediatric patient with allergy to penicillins able to take oral medication	Amoxicillin	1 (1%)	0 (0%)	0 (0%)	1 (3%)	0.03
	Azithromycin or clarithromycin	41 (45%)	40 (53%)	23 (59%)	13 (36%)	
	Cephalosporin	3 (3%)	5 (7%)	1 (3%)	5 (14%)	
	Cefazolin or ceftriaxone	0 (0%)	0 (0%)	0 (0%)	1 (3%)	
	Clindamycin	31 (34%)	19 (25%)	12 (31%)	5 (14%)	

	I don't know	16 (17%)	11 (15%)	3 (8%)	11 (31%)	
Dosage *(correct antibiotic)*	High	6 (8%)	6 (9%)	7 (19%)	7 (30%)	0.15
	Low	32 (43%)	24 (38%)	15 (42%)	10 (43%)	
	Right	19 (25%)	20 (31%)	8 (22%)	2 (9%)	
	I don't know	18 (24%)	14 (22%)	6 (17%)	4 (17%)	
Timing *(correct antibiotic)*	Double dose: half 30-60 minutes before the procedure + half 30-60 minutes after the procedure	17 (23%)	10 (16%)	4 (11%)	4 (17%)	0.18
	I don't know	9 (12%)	6 (9%)	1 (3%)	0 (0%)	
	Single dose 30-60 minutes after procedure	5 (7%)	2 (3%)	5 (14%)	1 (4%)	
	Single dose 30-60 minutes before procedure	44 (59%)	46 (72%)	26 (72%)	18 (78%)	

Table S14. Knowledge on the antibiotic of first choice for the prophylaxis of IE in patients with no allergy to penicillins on the basis of academic level.

**Variable**	**Category**	**General dentist (n=141)**	**Orthodontics or Oral surgery specialist (n=77)**	**Pediatric dentistry specialist (n=24)**	**p-value**
Antibiotic of first choice for the prophylaxis of IE in pediatric patient with allergy to penicillins able to take oral medication	Amoxicillin	1 (1%)	1 (1%)	0 (0%)	0.61
	Azithromycin or clarithromycin	67 (48%)	39 (51%)	11 (46%)	
	Cephalosporin	8 (6%)	5 (6%)	1 (4%)	
	Cefazolin or ceftriaxone	1 (1%)	0 (0%)	0 (0%)	
	Clindamycin	38 (27%)	18 (23%)	11 (46%)	
	I don't know	26 (18%)	14 (18%)	1 (4%)	
Dosage *(correct antibiotic)*	High	12 (11%)	10 (16%)	4 (17%)	0.75
	Low	46 (41%)	25 (40%)	10 (43%)	
	Right	31 (27%)	12 (19%)	6 (26%)	
	I don't know	24 (21%)	15 (24%)	3 (13%)	
Timing *(correct antibiotic)*	Double dose: half 30-60 minutes before the procedure + half 30-60 minutes after the procedure	24 (21%)	7 (11%)	4 (17%)	0.78
	I don't know	8 (7%)	6 (10%)	2 (9%)	

	Single dose 30-60 minutes after procedure	8 (7%)	4 (6%)	1 (6%)	
	Single dose 30-60 minutes before procedure	73 (65%)	45 (73%)	16 (70%)	

Table S15. Knowledge on the antibiotic of first choice for the prophylaxis of IE in patients with no allergy to penicillins on the basis of mainly treated population.

Variable	Category	Adults (n=32)	Adults and children/teenagers (n=138)	Children/teenagers (n=72)	p-value
Antibiotic of first choice for the prophylaxis of IE in pediatric patient with allergy to penicillins able to take oral medication	Amoxicillin	0 (0%)	1 (1%)	1 (1%)	0.31
	Azithromycin or clarithromycin	20 (63%)	59 (43%)	38 (53%)	
	Cephalosporin	0 (0%)	12 (9%)	2 (3%)	
	Cefazolin or ceftriaxone	0 (0%)	0 (0%)	1 (1%)	
	Clindamycin	8 (25%)	40 (29%)	19 (26%)	
	I don't know	4 (13%)	26 (19%)	11 (15%)	
Dosage *(correct antibiotic)*	High	3 (11%)	16 (14%)	7 (12%)	0.98
	Low	12 (43%)	44 (40%)	25 (42%)	
	Right	8 (29%)	26 (23%)	15 (25%)	
	I don't know	5 (18%)	25 (23%)	12 (20%)	
Timing *(correct antibiotic)*	Double dose: half 30-60 minutes before the procedure + half 30-60 minutes after the procedure	5 (18%)	22 (20%)	8 (14%)	0.11
	I don't know	1 (4%)	5 (5%)	10 (17%)	
	Single dose 30-60 minutes after procedure	2 (7%)	9 (8%)	2 (3%)	
	Single dose 30-60 minutes before procedure	20 (71%)	75 (68%)	39 (66%)	

Table S16. Knowledge on the antibiotic of first choice for the prophylaxis of IE in patients with no allergy to penicillins on the basis of sector of dental profession practicing.

Variable	Category	Private (n=187)	Public and public/private (n=55)	p-value
Antibiotic of first choice for the prophylaxis of IE in pediatric patient with allergy to penicillins able to take oral medication	Amoxicillin	1 (1%)	1 (2%)	0.41
	Azithromycin or clarithromycin	94 (50%)	23 (42%)	
	Cephalosporin	11 (6%)	3 (5%)	
	Cefazolin or ceftriaxone	1 (1%)	0 (0%)	
	Clindamycin	49 (26%)	18 (33%)	
	I don't know	31 (17%)	10 (18%)	
Dosage *(correct antibiotic)*	High	19 (12%)	7 (16%)	0.33
	Low	59 (38%)	22 (50%)	
	Right	40 (26%)	9 (20%)	
	I don't know	36 (23%)	6 (14%)	
Timing *(correct antibiotic)*	Double dose: half 30-60 minutes before the procedure + half 30-60 minutes after the procedure	25 (16%)	10 (23%)	0.56
	I don't know	14 (9%)	2 (5%)	
	Single dose 30-60 minutes after procedure	11 (7%)	2 (5%)	
	Single dose 30-60 minutes before procedure	104 (68%)	30 (68%)	
